# Sex differences in the association between socioeconomic status and diabetes prevalence and incidence in China: cross-sectional and prospective studies of 0.5 million adults

**DOI:** 10.1007/s00125-019-4896-z

**Published:** 2019-05-31

**Authors:** Hongjiang Wu, Fiona Bragg, Ling Yang, Huaidong Du, Yu Guo, Caroline A. Jackson, Shankuan Zhu, Canqing Yu, Andrea O. Y. Luk, Juliana C. N. Chan, Danijela Gasevic, Liming Li, Zhengming Chen, Sarah H. Wild

**Affiliations:** 1 Usher Institute of Population Health Sciences and Informatics, University of Edinburgh, Teviot Place, Edinburgh, EH8 9AG UK; 2 Nuffield Department of Population Health, University of Oxford, Oxford, UK; 3 Medical Research Council Population Health Research Unit at the University of Oxford, Oxford, UK; 4 Chinese Academy of Medical Sciences, Beijing, China; 5 School of Public Health, Zhejiang University, Hangzhou, China; 6 Department of Epidemiology and Biostatistics, School of Public Health, Peking University Health Science Center, Beijing, China; 7 Department of Medicine and Therapeutics, The Chinese University of Hong Kong, Prince of Wales Hospital, Shatin, Hong Kong China; 8 School of Public Health and Preventive Medicine, Monash University, Melbourne, VIC Australia

**Keywords:** Diabetes, Educational level, Health inequality, Household income, Socioeconomic status

## Abstract

**Aims/hypothesis:**

China has undergone rapid socioeconomic transition accompanied by lifestyle changes that are expected to have a profound impact on the health of its population. However, there is limited evidence from large nationwide studies about the relevance of socioeconomic status (SES) to risk of diabetes. We describe the associations of two key measures of SES with prevalent and incident diabetes in Chinese men and women.

**Methods:**

The China Kadoorie Biobank study included 0.5 million adults aged 30–79 years recruited from ten diverse areas in China during 2004–2008. SES was assessed using the highest educational level attained and annual household income. Prevalent diabetes was identified from self-report and plasma glucose measurements. Incident diabetes was identified from linkage to disease and death registries and national health insurance claim databases. We estimated adjusted ORs and HRs for prevalent and incident diabetes associated with SES using logistic and Cox regression models, respectively.

**Results:**

At baseline, 30,066 (5.9%) participants had previously diagnosed (3.1%) or screen-detected (2.8%) diabetes among 510,219 participants included for cross-sectional analyses. There were 480,153 people without prevalent diabetes at baseline, of whom 9544 (2.0%) had new-onset diabetes during follow-up (median 7 years). Adjusted ORs (95% CIs) for prevalent diabetes, comparing highest vs lowest educational level, were 1.21 (1.09, 1.35) in men and 0.69 (0.63, 0.76) in women; for incident diabetes, the corresponding HRs were 1.27 (1.07, 1.51) and 0.80 (0.67, 0.95), respectively. For household income, the adjusted ORs for prevalent diabetes, comparing highest vs lowest categories, were 1.45 (1.34, 1.56) in men and 1.26 (1.19, 1.34) in women; for incident diabetes, the HRs were 1.36 (1.19, 1.55) and 1.06 (0.95, 1.17), respectively.

**Conclusions/interpretation:**

Among Chinese adults, the associations between education and diabetes prevalence and incidence differed qualitatively between men and women, whereas higher household income was positively associated with diabetes prevalence and incidence in both sexes, with a stronger relationship in men than in women.

**Electronic supplementary material:**

The online version of this article (10.1007/s00125-019-4896-z) contains peer-reviewed but unedited supplementary material, which is available to authorised users.

## Introduction

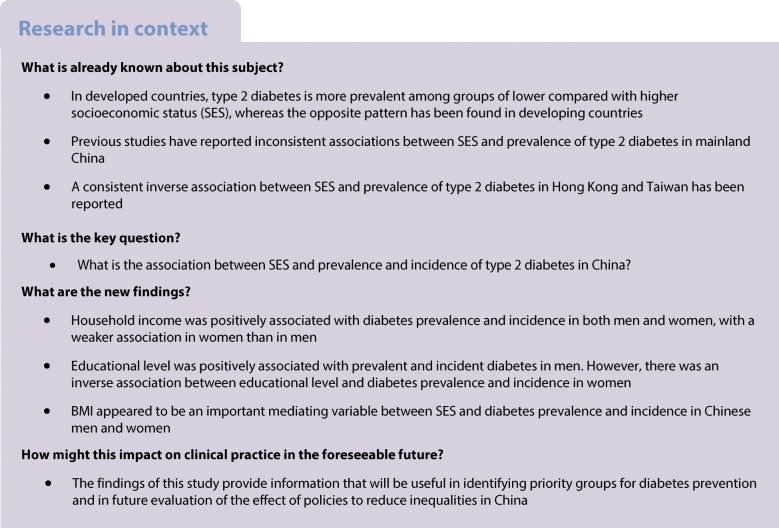



The prevalence of diabetes in China has increased markedly in the past few decades. The proportion of Chinese adults estimated to have diabetes was 0.9% in 1980, 2.5% in 1994 and 10.9% in 2013 [1–3]. The increase is thought to be the most rapid worldwide, and it is related to China’s recent rapid economic development and urbanisation, which are contributing to socioeconomic and epidemiological transition [4].

Evidence from developed countries that have completed the epidemiological transition shows that non-communicable diseases (NCDs) are initially more common in population subgroups of high socioeconomic status (SES) and then, with increasing development, become more common in lower SES groups [5]. However, the evidence from low- and middle-income countries (LMIC) is limited [6]. Previous studies have reported inconsistent associations between SES and diabetes prevalence in mainland China, and findings were not obviously influenced by study year or the level of economic development of the study area [7, 8]. However, in Hong Kong and Taiwan, where economic development and epidemiological transition are at a more advanced stage than in mainland China, an inverse association between SES and diabetes prevalence has been described [7]. Reliable assessment of the association between SES and diabetes in different parts of China is needed to plan and evaluate health services and diabetes prevention strategies. In order to examine the recent socioeconomic pattern of diabetes risk in China, we describe the associations between SES and both prevalent and incident diabetes, using data from the China Kadoorie Biobank (CKB), a large prospective cohort study of about 0.5 million Chinese adults.

## Methods

### Study population

Detailed information about the study design, survey methods and population of the CKB has been reported previously [9]. Briefly, the baseline survey took place between June 2004 and July 2008 in ten geographically defined areas (five urban and five rural) of China. The areas were selected according to local disease patterns, exposure to certain risk factors, population stability, levels of SES, quality of death and disease registries, local commitment and capacity. Overall, 512,891 adults aged 30–79 years were enrolled, with a response rate of about 30%.

Ethical approval for this study was obtained from the University of Oxford, the Chinese Centre for Disease Control and Prevention and the local Centres for Disease Control and Prevention in the ten study regions. All participants provided written informed consent.

### Assessment of socioeconomic status

We assessed participants’ SES status in two ways: (1) the highest level of school education an individual attained (no formal school, primary school, middle or high school, and college or above); and (2) total household income in the previous year (<10,000, 10,000–19,999, 20,000–34,999, and ≥35,000 Chinese yuan).

### Assessment of covariates

We obtained covariates from the baseline questionnaire, including demographic characteristics (age, sex, urban and rural residence, and study regions), health-related behaviours (regular active and passive smoking, regular consumption of alcohol, fresh fruit, fresh vegetables and fish), personal and family medical history (history of CHD, stroke or transient ischaemic attack [TIA], cancer and diabetes). Regular active smoking was defined as current smoking on most days or more. Regular passive smoking was defined as exposure to other people’s tobacco smoke for 4 or more days per week. Regular consumption of alcohol was defined as drinking alcohol monthly or more frequently. Regular consumption of fresh fruit, vegetables and fish was defined as consumption of these foods on four or more days per week. A range of physical measurements were undertaken by trained technicians using a standard protocol and calibrated instruments, including BMI, waist and hip circumference, fat percentage and systolic and diastolic BP. Physical activity was estimated by summing the metabolic equivalent task (MET) h per day spent on work, commuting, housework and non-sedentary recreational activities [10]. Economic–geographic areas of China were categorised into four groups according to the National Bureau of Statistics of China and ranked from high to low per capita disposable income of households as Eastern, Northeastern, Central and Western, which reflected different levels of economic development in China [11]. For BMI, we used cut-off points for Chinese populations to define overweight (≥24 kg/m^2^ and <28 kg/m^2^) and obesity (≥28 kg/m^2^) [12].

### Baseline prevalent diabetes, follow-up and ascertainment of incident diabetes

A 10 ml non-fasting blood sample was collected from study participants at baseline and fasting time was recorded. Plasma glucose levels were tested on-site using the SureStep Plus meter (LifeScan, Shanghai, China). Participants with plasma glucose levels from 7.8 mmol/l to less than 11.1 mmol/l were invited to return for a fasting plasma glucose test on the following day. Participants were asked at baseline: ‘Has a doctor ever told you that you had diabetes?’, with those reporting ‘yes’ defined as having self-reported previously diagnosed diabetes. Screen-detected diabetes was defined among participants without self-reported diabetes on the basis of any of: (1) random plasma glucose level ≥7.0 mmol/l and a fasting time ≥8 h; (2) random plasma glucose level ≥11.1 mmol/l and a fasting time <8 h; and (3) fasting plasma glucose level ≥7.0 mmol/l. Prevalent diabetes includes both self-reported previously diagnosed diabetes and screen-detected diabetes.

The vital status of participants was obtained periodically from local death registries based at China’s Disease Surveillance Points system, checked annually against local residential records and health insurance records and confirmed with street committees or village administrators. Incident diabetes was identified from linkage to disease and death registries and national health insurance databases, collecting details of diagnoses resulting in, or during, any hospital admission via individuals’ unique national ID. We defined incident diabetes using the E10-E14 codes from the Tenth Revision of the International Classification of Diseases (http://apps.who.int/classifications/icd10/browse/2016/en).

### Statistical analysis

**Fig. 1:**
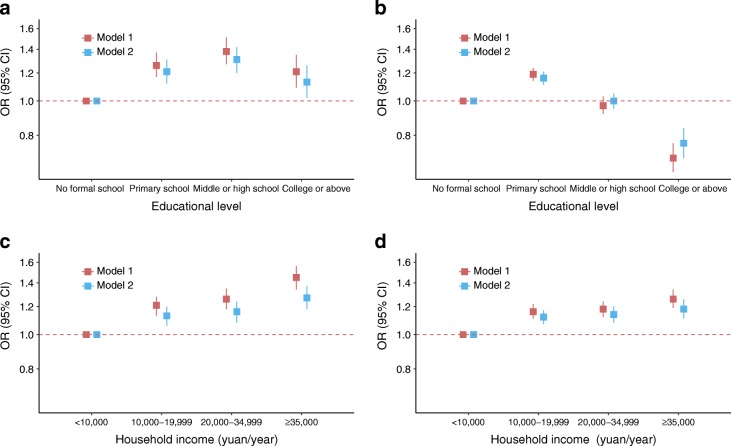
Adjusted ORs and 95% CIs for prevalent diabetes associated with educational level (**a**, **b**) and household income (**c**, **d**) in men (**a**, **c**) and women (**b**, **d**), plotted on a logarithmic scale. For educational level, model 1 was adjusted for age at baseline (continuous), study region, family history of diabetes and household income. Model 2 was further adjusted for BMI based on model 1. For household income, model 1 was adjusted for age at baseline (continuous), study region, family history of diabetes, educational level and household size. Model 2 was further adjusted for BMI based on model 1. See ESM Tables 3 and 4 for data

We excluded 2672 (0.5%) participants with missing, implausible or extreme values for all variables, leaving 510,219 participants for cross-sectional analyses of prevalent diabetes (electronic supplementary material [ESM] Fig. 1). We excluded 30,066 (5.9%) participants with previously diagnosed diabetes (3.1%) or screen-detected diabetes (2.8%) at baseline, leaving 480,153 participants for prospective analyses of incident diabetes. All analyses were stratified by sex. Logistic regression was used to estimate ORs and 95% CIs for the association between SES and baseline prevalent diabetes. For educational level, we adjusted for confounding variables including age at baseline (continuous), ten study regions, family history of diabetes, and household income in model 1. To further assess potential mediating variables for the association of SES with diabetes, we added BMI as a continuous variable to model 1 to create model 2. In model 3, we added other potential mediating variables, including waist circumference, fat percentage, physical activity, regular alcohol consumption, regular active smoking, regular passive smoking, consumption of fresh fruit, fresh vegetables and fish, history of CHD, stroke or TIA and cancer, systolic BP and diastolic BP. The potential mediating variables were selected based on prior knowledge of underlying mechanisms linking SES and diabetes [13, 14]. For household income, we built similar models but further adjusted for educational level and household size in all models. There was no evidence of serious multicollinearity in any model based on values of variance inflation factors.

To estimate incidence, we calculated person-years from baseline until the date of first record of incident diabetes, death, loss to follow-up or 31 December 2013, whichever came first. We used stratified Cox regression models to estimate the HRs and 95% CIs for the association between SES and incident diabetes, with various models constructed in similar ways to those used for analysing prevalent diabetes. We checked the Cox proportional hazards assumption using Schoenfeld residuals and found the assumption was not violated for any model. We performed likelihood ratio tests to investigate potential interactions between SES and variables of interest, by comparing models with and without interaction terms, and investigated potential linear trend effects of SES on incident diabetes by comparing models including and excluding a linear effect term.

We performed further analyses to investigate the impact of a composite measure of educational level and household income on incident diabetes. We categorised participants into four groups: (1) low educational level and low income; (2) low educational level and high income; (3) high educational level and low income; and (4) high educational level and high income. Low educational level was defined as having education at primary school level or below; low household income was defined as having annual household income <20,000 Chinese yuan. Additional sensitivity analyses were done after excluding individuals with baseline CHD, stroke or TIA, or cancer, to reduce potential reverse causality. R software (version 3.3.3; www.R-project.org, Vienna, Austria) was used to perform the analyses.

## Results

### Characteristics of participants and patterns of covariates with SES

Among 510,219 participants included for cross-sectional analyses, 59.0% were women and 44.1% were from urban areas (Table 1). The mean (SD) age was 52.3 (10.9) years for men and 50.9 (10.5) years for women. Higher proportions of men than women received college-level education or above (7.9% vs 4.5%) and were in the highest household income group (20.3% vs 16.5%), and lower proportions of men than women received no formal education (8.9% vs 25.2%) and were in the lowest household income group (26.0% vs 29.7%). Men with higher educational level and household income were more likely to be overweight or obese and had higher BMI, waist circumference and percentage fat than those in lower SES groups (ESM Tables 1 and 2). The absolute differences in these covariates by SES in women were very small, with values slightly higher in both the middle educational level and the middle household income groups compared with those at either extreme (ESM Tables 1 and 2).

**Table 1:** Sex-specific characteristics of CKB participants included in cross-sectional analyses

Variable	Men (*n* = 209,352)	Women (*n* = 300,867)	All (*n* = 510,219)
Age, years	52.3 (10.9)	50.9 (10.5)	51.5 (10.7)
Urban residence, *n* (%)	90,936 (43.4)	133,913 (44.5)	224,849 (44.1)
Geographic area, *n* (%)			
Eastern	72,531 (34.6)	102,692 (34.1)	175,233 (34.3)
Northeastern	23,170 (11.1)	34,164 (11.4)	57,334 (11.2)
Central	54,038 (25.8)	68,757 (22.9)	122,795 (24.1)
Western	59,613 (28.5)	95,254 (31.7)	154,867 (30.4)
Educational level, *n* (%)			
No formal school	18,529 (8.9)	75,834 (25.2)	94,363 (18.5)
Primary school	69,769 (33.3)	94,571 (31.4)	164,340 (32.2)
Middle or high school	104,569 (49.9)	117,037 (38.9)	221,606 (43.4)
College or above	16,485 (7.9)	13,425 (4.5)	29,910 (5.9)
Household income (yuan/year), *n* (%)			
<10,000	54,386 (26.0)	89,437 (29.7)	143,823 (28.2)
10,000–19,999	59,335 (28.3)	88,959 (29.6)	148,294 (29.1)
20,000–34,999	53,229 (25.4)	72,911 (24.2)	126,140 (24.7)
≥35,000	42,402 (20.3)	49,560 (16.5)	91,962 (18.0)
Regular alcohol consumption, *n* (%)	93,142 (44.5)	11,738 (3.9)	104,880 (20.6)
Regular smoking, *n* (%)			
Active	127,862 (61.1)	7103 (2.4)	134,965 (26.5)
Passive	116,683 (55.7)	156,950 (52.2)	273,633 (53.6)
Regular consumption of foods, *n* (%)			
Fresh fruit	48,237 (23.0)	95,693 (31.8)	143,930 (28.2)
Fresh vegetables	205,726 (98.3)	295,846 (98.3)	501,572 (98.3)
Fish	19,820 (9.5)	25,499 (8.5)	45,319 (8.9)
Prior diseases, *n* (%)			
CHD	5683 (2.7)	9654 (3.2)	15,337 (3.0)
Stroke or TIA	4873 (2.3)	3926 (1.3)	8799 (1.7)
Cancer	963 (0.46)	1599 (0.53)	2562 (0.50)
Family history of diabetes, *n* (%)	9894 (4.7)	15,130 (5.0)	25,024 (4.9)
BMI (kg/m^2^)			
Mean	23.4 (3.2)	23.8 (3.4)	23.7 (3.4)
<24 (normal), *n* (%)	122,143 (58.3)	164,643 (54.7)	286,786 (56.2)
24–28 (overweight), *n* (%)	68,628 (32.8)	100,927 (33.5)	169,555 (33.2)
≥28 (obese), *n* (%)	18,581 (8.9)	35,297 (11.7)	53,878 (10.6)
Physical activity, MET-h/day	22.0 (15.3)	20.4 (12.8)	21.1 (13.9)
Systolic BP, mmHg	132.8 (20.0)	129.8 (21.9)	131.1 (21.2)
Diastolic BP, mmHg	79.2 (11.3)	76.8 (10.9)	77.8 (11.1)
Waist circumference, cm	82.0 (9.7)	79.1 (9.5)	80.3 (9.7)
Fat, %	22.0 (6.2)	32.1 (7.1)	30.0 (8.4)

Values are shown as *n* (%) or mean (SD)

### Associations between SES and prevalent diabetes

Age-standardised diabetes prevalence increased with higher educational level and household income in men, but the patterns were less clear in women (Table 2). After adjustment for age, study region, family history of diabetes and household income, there was a positive association between educational level and prevalent diabetes in men and an inverse association in women (*p*_interaction_ < 0.0001 between sex) (Fig. 1). The ORs (95% CIs) derived from model 1, comparing highest vs lowest educational level, were 1.21 (1.09, 1.35) in men and 0.69 (0.63, 0.76) in women. There was a positive association between household income and prevalent diabetes in both men and women, with the association more pronounced in men than in women (*p*_interaction_ < 0.0001 between sex) (Fig. 1). The ORs (95% CIs), comparing highest vs lowest household income category from model 1, were 1.45 (1.34, 1.56) in men and 1.26 (1.19, 1.34) in women. These associations were attenuated after additional adjustment for BMI but remained statistically significant, changing slightly after further adjustment for other potential mediating variables (Fig. 1, ESM Tables 3 and 4).

**Table 2:** Sex-specific diabetes prevalence among CKB participants included in cross-sectional analyses according to educational level and household income

Socioeconomic status	Men (*n* = 209,352)		Women (*n* = 300,867)	
	No. of participants with prevalent diabetes	Standardised prevalence rate,^a^ % (95% CI)	No. of participants with prevalent diabetes	Standardised prevalence rate,^a^ % (95% CI)
All	11,616	5.55 (5.45, 5.65)^b^	18,450	6.13 (6.04, 6.22)^b^
Educational level				
No formal school	863	3.55 (3.47, 3.64)	5523	5.47 (5.30, 5.64)
Primary school	3444	4.24 (4.15, 4.33)	6230	6.17 (6.01, 6.33)
Middle and high school	5950	6.91 (6.81, 7.02)	6033	7.45 (7.24, 7.67)
College or above	1359	9.03 (8.91, 9.15)	664	6.97 (6.43, 7.54)
Household income, yuan/year				
<10,000	2232	3.86 (3.77, 3.95)	5037	5.27 (5.19, 5.34)
10,000–19,999	3400	5.75 (5.55, 5.94)	5802	6.65 (6.48, 6.82)
20,000–34,999	3238	6.31 (6.09, 6.53)	4544	6.61 (6.42, 6.81)
≥35,000	2746	6.94 (6.68, 7.21)	3067	6.49 (6.26, 6.73)

^a^5 year age group standardised to the whole CKB population

^b^Crude prevalence

No., number

### Associations between SES and incident diabetes

**Fig. 2:**
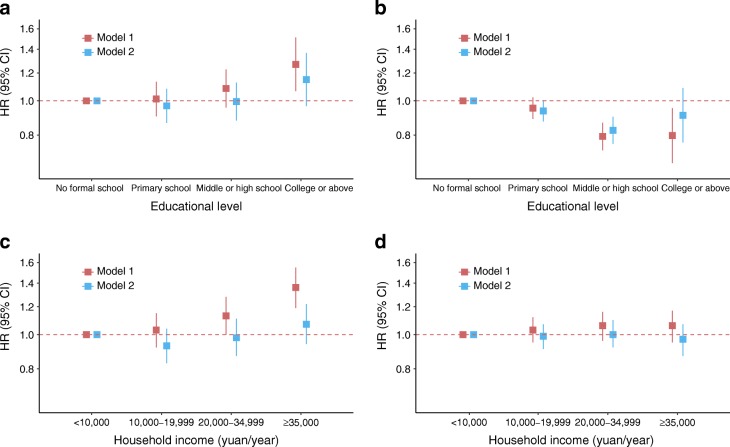
Adjusted HRs and 95% CIs for incident diabetes associated with educational level (**a**, **b**) and household income (**c**, **d**) in men (**a**, **c**) and women (**b**, **d**), plotted on a logarithmic scale. For educational level, model 1 was stratified by age at baseline (5 year age group) and study region, and adjusted for age at baseline (continuous), family history of diabetes and household income. Model 2 was further adjusted for BMI based on model 1. For household income, model 1 was stratified by age at baseline (5 year age group) and study region, and adjusted for age at baseline (continuous), family history of diabetes, educational level and household size. Model 2 was further adjusted for BMI based on model 1. See ESM Tables 8 and 9 for data

The characteristics of participants without diabetes at baseline (*n* = 480,153) by SES were similar to those of people included for cross-sectional analyses (ESM Tables 5–7). During a median of 7 years (3.4 million person-years total) of follow-up, 2032 (0.4%) participants were lost to follow-up and 22,103 (4.6%) participants died. In total, 9544 new cases of diabetes were identified, 5937 among women (Table 3). Age-standardised diabetes incidence increased with higher household income in both men and women. A U-shaped association was observed between educational level and diabetes incidence because the lowest incidence of diabetes occurred in both men and women who had received a maximum of middle or high school education (Table 3). The associations of SES with incident diabetes were similar to those with prevalent diabetes. In model 1, educational level was positively associated with incident diabetes in men, but an inverse association was found in women (*p*_interaction_ < 0.0001 between sex) (Fig. 2). The adjusted HRs (95% CIs), comparing highest vs lowest educational level, were 1.27 (1.07, 1.51) in men and 0.80 (0.67, 0.95) in women. Household income was also positively associated with incident diabetes in men, but there was a non-significant positive association between household income and incident diabetes in women (*p*_interaction_ < 0.0001 between sexes) (Fig. 2). The adjusted HRs (95% CIs), comparing highest vs lowest household income category, were 1.36 (1.19, 1.55) in men and 1.06 (0.95, 1.17) in women. There was no evidence of a departure from linear trend for any analysis (*p* ≥ 0.05), except for educational level and incident diabetes in women (*p* = 0.047). After additional adjustment for BMI, the associations between SES and incident diabetes were markedly attenuated and no longer statistically significant at the 5% level for any comparison between the highest and lowest SES groups (Fig. 2, ESM Tables 8 and 9). Further adjustment for other potential mediating factors had little effect on the results (ESM Tables 8 and 9).

**Table 3:** Sex-specific diabetes incidence among CKB participants included in prospective analyses according to educational level and household income

Socioeconomic status	Men (*n* = 197,736)		Women (282,417)	
	No. of participants with incident diabetes	Standardised incidence rate^a^ (no./1000 person-years)	No. of participants with incident diabetes	Standardised incidence rate^a^ (no./1000 person-years)
All	3607	2.59 (2.51, 2.68)^b^	5937	2.93 (2.86, 3.01)^b^
Highest educational level				
No formal school	480	3.38 (2.98, 3.85)	2330	3.80 (3.62, 3.99)
Primary school	1342	2.65 (2.49, 2.81)	1945	2.96 (2.83, 3.10)
Middle or high school	1469	2.37 (2.25, 2.51)	1485	2.29 (2.16, 2.43)
College or above	316	3.24 (2.88, 3.64)	177	2.51 (2.13, 2.94)
Household income, yuan/year				
<10,000	747	1.86 (1.73, 2.00)	1522	2.36 (2.23, 2.48)
10,000–19,999	835	2.11 (1.97, 2.25)	1536	2.59 (2.46, 2.72)
20,000–34,999	1003	2.95 (2.77, 3.14)	1650	3.49 (3.32, 3.67)
≥35,000	1022	3.89 (3.65, 4.14)	1229	3.87 (3.65, 4.09)

^a^5 year age group standardised to the whole CKB population

^b^Crude incidence

No., number

The association between educational level and incident diabetes did not appear to be modified by age, urban or rural residence, economic–geographic areas or household income in men or women (ESM Fig. 2). However, the positive association of household income with incident diabetes was more pronounced in urban areas than in rural areas in men (*p*_interaction_ = 0.035) but not in women (*p*_interaction_ = 0.99) and was more pronounced in less economically developed geographic areas (Northeastern, Central and Western) than in more developed areas (Eastern) in both sexes (*p*_interaction_ = 0.017 in men and *p*_interaction_ = 0.010 in women) (ESM Fig. 3). Absolute risk of incident diabetes was lowest in men with low household income regardless of educational level and highest in women with low educational level and high household income (ESM Table 10). The relative risk of developing diabetes compared with the groups with low educational level and low household income was highest in men who had both high educational level and high household income and in women who had low educational level but high household income (ESM Table 10). Excluding people with a history of any of CHD, stroke, TIA or cancer at baseline from the study population had no effect on the associations of SES with prevalent or incident diabetes (ESM Tables 11 and 12).

## Discussion

This large nationwide prospective study in about 0.5 million Chinese adults showed that the associations between SES and diabetes prevalence and incidence differ between men and women. Among men, both educational level and household income were positively associated with risk of diabetes. Among women, there was an inverse association between educational level and risk of diabetes, whereas for household income the positive associations with diabetes prevalence and incidence were weaker than in men. The associations of educational level and household income with diabetes appeared to be partly mediated by BMI.

A previous systematic review reported inconsistent associations of educational level and income with prevalent type 2 diabetes in China [7]. Most of the studies included in the systematic review had small sample sizes, were restricted to a particular geographic area and the association between SES and diabetes prevalence was rarely the primary research question. Few previous studies explored the association separately in men and women.

To our knowledge, our study is the first nationwide prospective study to describe the association between SES and diabetes incidence in China. The positive association between SES and incident diabetes in men in our study contrasts with findings from high-income countries [15] and Taiwan [16], but they are consistent with those from Thailand [17]. Evidence for an association between SES and incident diabetes from other LMIC is sparse [15]. A study of 10,704 Chinese adults living in Qingdao, a coastal city in China, reported an inverse association between educational level and incident diabetes in both sexes during 2001–11 [18]. In that study, a high proportion of both men and women had college education or higher (16.8% and 24.0%, respectively) as opposed to 7.9% and 4.5% in the present study and 8.7% of men and 6.1% of women in similar age groups in the 2010 Chinese census [19]. It is possible that the Qingdao findings represent the patterns of association in Chinese populations at a more advanced stage of epidemiological transition than other parts of China. The proportions of people in the highest education category in our study were similar to those of participants in the 2010 Chinese census. However, it is important to recognise that the patterns we observed may not reflect those in the whole population of China; the CKB sample was never intended to be nationally representative and the response rate was approximately 30% (comparing favourably with other large nationwide biobank studies, such as the UK Biobank).

SES has profound effects on health through complex processes, such as access to healthcare, health behaviours and environmental exposures [20]. The mechanisms through which SES influences the development of diabetes are not fully understood. The association between SES and diabetes may be partially explained by the distribution of conventional risk factors for diabetes such as being overweight or obese, which are strongly patterned by SES in many populations [13, 14]. In this study, the associations between SES and both diabetes prevalence and incidence were attenuated after adjustment for BMI, suggesting that BMI was likely to be a key mediator in the pathway linking SES and diabetes prevalence and incidence in the Chinese population. This finding was consistent with that from western countries [21].

We observed sex differences in the associations between educational level and diabetes prevalence and incidence. These sex differences can be at least partly attributed to differences in the relationship between education and BMI in men and women. Previous cross-sectional studies in China have reported that men with higher educational level were more likely to be overweight or obese than those of lower educational attainment, while the converse was true in Chinese women [22–24]. A review of studies from LMIC reported that the burden of obesity tends to shift from groups with high to low educational level during economic development, and this transition occurs earlier for women than men [25]. This evidence may explain the inconsistent findings between men and women in our study. Further exploration of the association between SES and BMI in China would be interesting but was beyond the scope of this study.

Interestingly, in women, we observed an inverse association between educational level and incident diabetes, while the association between household income and incident diabetes was positive (although not statistically significant). Compared with income, educational level is probably more strongly linked to an individual’s cognitive functioning and health-promoting behaviours, and plays a greater role in the onset of diseases, while income is more strongly related to the progression of diseases [26]. This suggests that educational level may be a more sensitive predictor of disease development than income during the epidemiological transition, at least in women. We found some evidence that the positive association between income and incident diabetes was weaker in both sexes in more economically developed geographic areas than in those at earlier stages of development. This may indicate that the transition in the association between SES and risk of diabetes from being positive to inverse is already occurring in more economically developed areas in China. However, more evidence is required to support this hypothesis.

### Strengths and limitations

A key strength of this study is that it is the first nationwide study to describe contemporary associations between SES and diabetes incidence in China. The large and diverse population sample permits the investigation of potential differences in the association between SES and diabetes in settings where economic development and epidemiological transition may be at different stages. Reviews of medical records in about 1000 incident cases identified a positive predictive value of 97% based on ADA diagnostic criteria [27] and medication use. In addition, extremely low loss to follow-up limits the potential for biased risk estimates.

One of the key study limitations is potential under-ascertainment of both prevalent and incident diabetes. The diabetes prevalence in the CKB was slightly lower than in contemporaneous nationally representative surveys in which diabetes diagnosis was based on a combination of self-report, fasting blood glucose, glycated haemoglobin measurement and oral glucose tolerance tests [2, 28]. It is not clear whether under-ascertainment of prevalent diabetes differed across SES groups. For incident diabetes, only hospitalised diabetes events were identified in the CKB. Hospital admission rates may be greatly affected by people’s ability to pay and vary by SES in China. If Chinese people of higher SES are more likely to be admitted to hospital, as reported by a previous study [29], then under-ascertainment of diabetes might be higher in lower SES groups; this would bias the association between SES and incident diabetes. We established that the proportion of prevalent diabetes that was self-reported increased with higher SES in both men and women in the CKB population. Our findings would therefore potentially have exaggerated the positive association between SES and incident diabetes in men, and the inverse association with educational level in women is likely to have been underestimated. Future research is required with more complete diabetes case ascertainment, such as from repeated cross-sectional measures of diabetes status using reliable laboratory tests. Furthermore, the statistical power of detecting a 6% relative difference in hazard of incident diabetes between the highest and lowest household income in women is low (power = 0.31), which may contribute to the non-significant positive association between household income and incident diabetes. We were not able to identify type of diabetes, but most diabetes cases were likely to be type 2 diabetes given the age of the study population.

In summary, we found that among Chinese adults in mainland China, educational level was positively associated with prevalent and incident diabetes in men, but inversely associated with diabetes in women. Household income was positively associated with prevalent and incident diabetes in both sexes. The findings of this study provide useful information for identifying priority groups for prevention of diabetes and to allow evaluation of the effect of policies that influence health inequalities in China. Health and social policies that aim to reduce socioeconomic and geographic inequalities in diabetes burden should give priority to primary and secondary prevention of overweight and obesity in Chinese adults and take account of potentially different associations with SES in men and women. Future research is needed to identify effective approaches to reducing inequalities by SES in diabetes incidence and to investigate whether SES also affects the risk of developing diabetic complications in China.

## Electronic supplementary material

ESM (PDF 1284 kb)

## Data Availability

Details of how to access the CKB data and details of the data release schedule are available from www.ckbiobank.org/site/Data+Access.

## Abbreviations

CKB: China Kadoorie Biobank
LMIC: Low- and middle-income countries
MET: Metabolic equivalent task
NCD: Non-communicable disease
SES: Socioeconomic status
TIA: Transient ischaemic attack
