# Supplementary material for: Sex differences in the association between socioeconomic status and diabetes prevalence and incidence in China: cross-sectional and prospective studies of 0.5 million adults
Source: Diabetologia. 2019 May 31;62(8):1420–9. doi: 10.1007/s00125-019-4896-z (PMC6647183; doi:10.1007/s00125-019-4896-z)
Supplement: Supplementary file 1 — (PDF 1284 kb) [file 125_2019_4896_MOESM1_ESM.pdf]

ESM Table 1. Sex specific characteristics of CKB participants included for cross-sectional analyses by educational level

Variable	Highest educational level attained								
	Men (n=209352)					Women (n=300867)			
	No formal school (n=18529)	Primary school (n=69769)	Middle or high school (n=104569)	College or above (n=16485)		No formal school (n=75834)	Primary school (n=94571)	Middle or high school (n=117037)	College or above (n=13425)
Age, years	60.3 (9.9)	55.9 (10.1)	48.8 (9.8)	50.8 (12.3)		56.8 (10.1)	51.8 (10.2)	46.8 (8.8)	46.7 (10.4)
Urban residence, *n* (%)	3576 (19.3)	17882 (25.6)	55058 (52.7)	14420 (87.5)		22989 (30.3)	27116 (28.7)	71455 (61.1)	12353 (92.0)
Geographic area, *n* (%)									
Eastern	9483 (51.2)	23220 (33.3)	34938 (33.4)	4890 (29.7)		37921 (50.0)	26628 (28.2)	34946 (29.9)	3197 (23.8)
Northeastern	310 (1.7)	1757 (2.5)	14671 (14.0)	6432 (39.0)		2077 (2.7)	3966 (4.2)	21586 (18.4)	6535 (48.7)
Central	1834 (9.9)	23619 (33.9)	27708 (26.5)	877 (5.3)		9432 (12.4)	33748 (35.7)	25158 (21.5)	419 (3.1)
Western	6902 (37.2)	21173 (30.3)	27252 (26.1)	4286 (26.0)		26404 (34.8)	30229 (32.0)	35347 (30.2)	3274 (24.4)
Household income (yuan/year), *n* (%)									
<10,000	8805 (47.5)	24475 (35.1)	20711 (19.8)	395 (2.4)		33431 (44.1)	32203 (34.1)	23518 (20.1)	285 (2.1)
10,000-19,999	3529 (19.0)	19974 (28.6)	33507 (32.0)	2325 (14.1)		16863 (22.2)	30735 (32.5)	39664 (33.9)	1697 (12.6)
20,000-34,999	3534 (19.1)	14756 (21.1)	29664 (28.4)	5275 (32.0)		14508 (19.1)	20512 (21.7)	33381 (28.5)	4510 (33.6)
≥35,000	2661 (14.4)	10564 (15.1)	20687 (19.8)	8490 (51.5)		11032 (14.5)	11121 (11.8)	20474 (17.5)	6933 (51.6)
Regular alcohol consumption, *n* (%)	6470 (34.9)	29160 (41.8)	49536 (47.7)	7976 (48.4)		2103 (2.8)	3350 (3.5)	5090 (4.3)	1195 (8.9)
Regular smoking, *n* (%)									
Active	11939 (64.4)	45200 (64.8)	64103 (61.3)	6620 (40.2)		2350 (3.1)	2881 (3.0)	1771 (1.5)	101 (0.75)
Passive	8654 (46.7)	39002 (55.9)	61270 (58.6)	7757 (47.1)		35008 (46.2)	53180 (56.2)	63122 (53.9)	5640 (42.0)
Regular consumption of foods, *n* (%)									
Fresh fruit	2204 (11.9)	9734 (14.0)	27623 (26.4)	8676 (52.6)		11671 (15.4)	19844 (21.0)	53624 (45.8)	10554 (78.6)
Fresh vegetable	17683 (95.4)	68612 (98.3)	103070 (98.6)	16361 (99.2)		73113 (96.4)	93316 (98.7)	116065 (99.2)	13352 (99.5)

Fish	914 (4.9)	4450 (6.4)	11787 (11.3)	2669 (16.2)		4797 (6.3)	6119 (6.5)	12778 (10.9)	1805 (13.4)
Prior diseases, *n* (%)									
CHD	376 (2.0)	1807 (2.6)	2575 (2.5)	925 (5.6)		1994 (2.6)	3108 (3.3)	3820 (3.3)	732 (5.5)
Stroke or TIA	465 (2.5)	1671 (2.4)	2230 (2.1)	507 (3.1)		1030 (1.4)	1347 (1.4)	1364 (1.2)	185 (1.4)
Cancer	98 (0.53)	385 (0.55)	383 (0.37)	97 (0.59)		348 (0.46)	519 (0.55)	615 (0.53)	117 (0.87)
Family history of diabetes, *n* (%)	239 (1.3)	1512 (2.2)	6646 (6.4)	1497 (9.1)		1298 (1.7)	2655 (2.8)	9312 (8.0)	1865 (13.9)
BMI (kg/m^2)									
Mean	22.3 (3.0)	22.9 (3.1)	23.8 (3.2)	24.7 (3.1)		23.7 (3.5)	23.9 (3.5)	23.9 (3.3)	23.3 (3.1)
<24 (normal), *n* (%)	13571 (73.2)	46172 (66.2)	55788 (53.4)	6612 (40.1)		42260 (55.7)	50864 (53.8)	63204 (54.0)	8315 (61.9)
24-28 (overweight), *n* (%)	4158 (22.4)	19200 (27.5)	37714 (36.1)	7556 (45.8)		24695 (32.6)	31906 (33.7)	40273 (34.4)	4053 (30.2)
≥28 (obese), *n* (%)	800 (4.3)	4397 (6.3)	11067 (10.6)	2317 (14.1)		8879 (11.7)	11801 (12.5)	13560 (11.6)	1057 (7.9)
Physical activity, Met-hr/day	23.7 (16.3)	22.2 (16.0)	22.6 (15.1)	15.6 (9.2)		21.9 (13.7)	20.2 (12.9)	20.0 (12.3)	17.9 (8.0)
Systolic blood pressure, mm Hg	138.5 (22.5)	134.7 (20.8)	131.0 (18.9)	130.0 (18.3)		137.8 (23.4)	132.0 (21.7)	124.3 (19.2)	117.8 (18.1)
Diastolic blood pressure, mm Hg	79.8 (11.5)	78.9 (11.3)	79.3 (11.4)	79.0 (11.0)		78.5 (11.1)	77.2 (10.8)	75.7 (10.6)	73.1 (10.2)
Waist circumference, cm	78.0 (9.2)	80.0 (9.5)	83.4 (9.6)	86.6 (8.9)		79.6 (10.1)	79.7 (9.7)	78.5 (8.9)	76.7 (8.3)
Fat, %	19.4 (6.0)	20.8 (6.0)	22.9 (6.1)	24.2 (5.7)		31.7 (7.5)	32.4 (7.3)	32.2 (6.7)	31.0 (6.2)

Values are shown as n (%) or mean (SD).

ESM Table 2. Sex specific characteristics of CKB participants included for cross-sectional analyses by household income

Variable	Household income (yuan/year)								
	Men (n=209352)					Women (n=300867)			
	<10000 (n=54386)	10000-19999 (n=95335)	20000-34999 (n=53229)	≥35000 (n=42402)		<10000 (n=89437)	10000-19999 (n=88959)	20000-34999 (n=72911)	≥35000 (n=49560)
Age, years	54.3 (11.4)	52.3 (10.9)	51.5 (10.5)	50.9 (10.4)		52.1 (11.4)	50.7 (10.3)	50.2 (9.8)	50.3 (9.8)
Urban residence, *n* (%)	9667 (17.8)	25099 (42.3)	29982 (56.3)	26188 (61.8)		20785 (23.2)	40652 (45.7)	41616 (57.1)	30860 (62.3)
Geographic area, *n* (%)									
Eastern	6817 (12.5)	14126 (23.8)	25842 (48.5)	25746 (60.7)		12715 (14.2)	22380 (25.2)	36401 (49.9)	31196 (62.9)
Northeastern	2140 (3.9)	7589 (12.8)	7651 (14.4)	5790 (13.7)		5134 (5.7)	11671 (13.1)	10286 (14.1)	7073 (14.3)
Central	15990 (29.4)	20728 (34.9)	11748 (22.1)	5572 (13.1)		19976 (22.3)	28145 (31.6)	14898 (20.4)	5738 (11.6)
Western	29439 (54.1)	16892 (28.5)	7988 (15.0)	5294 (12.5)		51612 (57.7)	26763 (30.1)	11326 (15.5)	5553 (11.2)
Highest educational level, *n* (%)									
No formal school	8805 (16.2)	3529 (5.9)	3534 (6.6)	2661 (6.3)		33431 (37.4)	16863 (19.0)	14508 (19.9)	11032 (22.3)
Primary school	24475 (45.0)	19974 (33.7)	14756 (27.7)	10564 (24.9)		32203 (36.0)	30735 (34.5)	20512 (28.1)	11121 (22.4)
Middle or high school	20711 (38.1)	33507 (56.5)	29664 (55.7)	20687 (48.8)		23518 (26.3)	39664 (44.6)	33381 (45.8)	20474 (41.3)
College or above	395 (0.73)	2325 (3.9)	5275 (9.9)	8490 (20.0)		285 (0.32)	1697 (1.9)	4510 (6.2)	6933 (14.0)
Regular alcohol consumption, *n* (%)	19831 (36.5)	26617 (44.9)	25589 (48.1)	21105 (49.8)		3431 (3.8)	3236 (3.6)	2820 (3.9)	2251 (4.5)
Regular smoking, *n* (%)									
Active	35132 (64.6)	35703 (60.2)	31827 (59.8)	25200 (59.4)		3547 (4.0)	2076 (2.3)	964 (1.3)	516 (1.0)
Passive	27528 (50.6)	33220 (56.0)	30571 (57.4)	25364 (59.8)		47484 (53.1)	47900 (53.8)	37112 (50.9)	24454 (49.3)
Regular consumption of foods, *n* (%)									
Fresh fruit	6195 (11.4)	12532 (21.1)	15087 (28.3)	14423 (34.0)		14836 (16.6)	27977 (31.4)	29203 (40.1)	23677 (47.8)
Fresh vegetable	52195 (96.0)	58642 (98.8)	52794 (99.2)	42095 (99.3)		85899 (96.0)	88201 (99.1)	72431 (99.3)	49315 (99.5)
Fish	1910 (3.5)	4497 (7.6)	6288 (11.8)	7125 (16.8)		3335 (3.7)	6577 (7.4)	7688 (10.5)	7899 (15.9)

Prior diseases, *n* (%)									
CHD	1088 (2.0)	1826 (3.1)	1615 (3.0)	1154 (2.7)		2367 (2.6)	3235 (3.6)	2453 (3.4)	1599 (3.2)
Stroke or TIA	1403 (2.6)	1583 (2.7)	1155 (2.2)	732 (1.7)		1208 (1.4)	1326 (1.5)	855 (1.2)	537 (1.1)
Cancer	295 (0.54)	262 (0.44)	220 (0.41)	186 (0.44)		444 (0.50)	508 (0.57)	405 (0.56)	242 (0.49)
Family history of diabetes, *n* (%)	1432 (2.6)	2740 (4.6)	3089 (5.8)	2633 (6.2)		2574 (2.9)	4527 (5.1)	4573 (6.3)	3456 (7.0)
BMI (kg/m^2)									
Mean	22.5 (3.1)	23.4 (3.2)	23.8 (3.2)	24.2 (3.2)		23.6 (3.5)	24.0 (3.5)	23.8 (3.4)	23.8 (3.3)
<24 (normal), *n* (%)	38331 (70.5)	34774 (58.6)	28559 (53.7)	20479 (48.3)		50967 (57.0)	46686 (52.5)	39977 (54.8)	27013 (54.5)
24-28 (overweight), *n* (%)	13239 (2.3)	19174 (32.3)	19291 (36.2)	16924 (39.9)		28368 (31.7)	30649 (34.5)	24772 (34.0)	17138 (34.6)
≥28 (obese), *n* (%)	2816 (5.2)	5387 (9.1)	5379 (10.1)	4999 (11.8)		10102 (11.3)	11624 (13.1)	8162 (11.2)	5409 (10.9)
Physical activity, Met-hr/day	21.8 (16.1)	21.2 (15.6)	23.0 (14.8)	22.4 (14.2)		20.4 (12.4)	19.0 (12.2)	21.5 (13.4)	21.6 (13.1)
Systolic blood pressure, mm Hg	133.6 (21.3)	132.4 (19.9)	132.7 (19.5)	132.4 (19.0)		132.0 (22.9)	129.4 (21.8)	129.0 (21.3)	128.1 (20.9)
Diastolic blood pressure, mm Hg	78.5 (11.4)	78.8 (11.4)	79.6 (11.3)	80.1 (11.1)		76.9 (11.2)	76.6 (10.9)	76.8 (10.7)	76.6 (10.5)
Waist circumference, cm	79.3 (9.1)	82.1 (9.8)	83.0 (9.8)	84.1 (9.7)		78.9 (9.7)	79.5 (9.7)	78.8 (92.5)	78.8 (89.2)
Fat, %	20.1 (6.0)	21.9 (6.1)	22.7 (6.1)	23.6 (6.1)		31.7 (7.3)	32.5 (7.1)	32.1 (6.9)	32.2 (6.7)

Values are shown as n (%) or mean (SD).

ESM Table 3. Adjusted ORs and 95% CIs for baseline prevalent diabetes associated with educational level in men and women

Educational level	Model 1		Model 2		Model 3	
	OR (95% CI)	p	OR (95% CI)	p	OR (95% CI)	p
Men						
No formal school	1		1		1	
Primary school	1.26 (1.17, 1.37)	<0.0001	1.21 (1.12, 1.31)	<0.0001	1.19 (1.10, 1.29)	<0.0001
Middle and high school	1.38 (1.27, 1.51)	<0.0001	1.31 (1.20, 1.42)	<0.0001	1.26 (1.15, 1.37)	<0.0001
College or above	1.21 (1.09, 1.35)	0.0003	1.13 (1.02, 1.26)	0.019	1.13 (1.02, 1.26)	0.024
p for departure for a linear trend[a]	<0.0001		<0.0001		<0.0001	
Women						
No formal school	1		1		1	
Primary school	1.19 (1.14, 1.24)	<0.0001	1.16 (1.11, 1.21)	<0.0001	1.16 (1.11, 1.22)	<0.0001
Middle and high school	0.97 (0.92, 1.03)	0.32	1.00 (0.95, 1.05)	0.90	1.11 (1.05, 1.17)	0.0001
College or above	0.69 (0.63, 0.76)	<0.0001	0.76 (0.69, 0.84)	<0.0001	0.96 (0.87, 1.06)	0.39
p for departure for a linear trend[a]	<0.0001		<0.0001		<0.0001	

Model 1: adjusted for age at baseline (continuous), study regions, family history of diabetes and household income.

Model 2: model 1 plus BMI (continuous).

Model 3: model 2 plus waist circumference, fat percentage, physical activity, regular alcohol consumption, active smoking, passive smoking, regular consumption of fish, regular consumption of fresh vegetable, regular consumption of fresh fruit, CHD, stroke or TIA, cancer, systolic blood pressure and diastolic blood pressure.

[a]Values≥0.05 suggests that the trend is linear.

ESM Table 4. Adjusted ORs and 95% CIs for baseline prevalent diabetes associated with household income in men and women

Household income (yuan/year)	Model 1		Model 2		Model 3	
	**OR (95% CI)**	**p**	**OR (95% CI)**	**p**	**OR (95% CI)**	**p**
Men						
<10,000	1		1		1	
10,000-19,999	1.21 (1.13, 1.28)	<0.0001	1.13 (1.06, 1.20)	0.0001	1.14 (1.07, 1.21)	<0.0001
20,000-34,999	1.26 (1.18, 1.35)	<0.0001	1.16 (1.08, 1.24)	<0.0001	1.18 (1.10, 1.26)	<0.0001
≥35,000	1.45 (1.34, 1.56)	<0.0001	1.27 (1.18, 1.37)	<0.0001	1.27 (1.17, 1.37)	<0.0001
p for departure for a linear trend[a]	0.01		0.12		0.12	
Women						
<10,000	1		1		1	
10,000-19,999	1.16 (1.11, 1.22)	<0.0001	1.12 (1.07, 1.17)	<0.0001	1.14 (1.09, 1.19)	<0.0001
20,000-34,999	1.18 (1.12, 1.24)	<0.0001	1.14 (1.08, 1.20)	<0.0001	1.19 (1.13, 1.25)	<0.0001
≥35,000	1.26 (1.19, 1.34)	<0.0001	1.18 (1.11, 1.26)	<0.0001	1.27 (1.19, 1.35)	<0.0001
p for departure for a linear trend[a]	0.0003		0.011		0.032	

Model 1: adjusted for age at baseline (continuous), study regions, family history of diabetes, educational level and household size.

Model 2: model 1 plus BMI (continuous).

Model 3: model 2 plus waist circumference, fat percentage, physical activity, regular alcohol consumption, active smoking, passive smoking, regular consumption of fish, regular consumption of fresh vegetable, regular consumption of fresh fruit, CHD, stroke or TIA, cancer, systolic blood pressure and diastolic blood pressure.

[a]Values≥0.05 suggests that the trend is linear.

ESM Table 5. Sex specific characteristics of CKB participants included for prospective analyses

Variable	Men (n=197736)	Women (n=282417)	All (n=480153)
Age, years	52.1 (10.9)	50.5 (10.4)	51.1 (10.6)
Urban residence, *n* (%)	83340 (42.1)	123233 (43.6)	206573 (43.0)
Geographic area, *n* (%)			
Eastern	68205 (34.5)	95879 (33.9)	164084 (34.2)
Northeastern	20563 (10.4)	30891 (10.9)	51454 (10.7)
Central	52374 (26.5)	65391 (23.2)	117765 (24.5)
Western	56594 (28.6)	90256 (32.0)	146850 (30.6)
Educational level, *n* (%)			
No formal school	17666 (8.9)	70311 (24.9)	87977 (18.3)
Primary school	66325 (33.5)	88341 (31.3)	154666 (32.2)
Middle or high school	98619 (49.9)	111004 (39.3)	209623 (43.7)
College or above	15126 (7.6)	12761 (4.5)	27887 (5.8)
Household income (yuan/year), *n* (%)			
<10,000	52154 (26.4)	84400 (29.9)	136554 (28.4)
10,000-19,999	55935 (28.3)	83157 (29.4)	139092 (29.0)
20,000-34,999	49991 (25.3)	68367 (24.2)	118358 (24.7)
≥35,000	39656 (20.1)	46493 (16.5)	86149 (17.9)
Regular alcohol consumption, *n* (%)	88168 (44.6)	271177 (4.0)	359345 (74.8)
Regular smoking, *n* (%)			
Active	122017 (61.7)	6509 (2.3)	128526 (26.8)
Passive	111131 (56.2)	148650 (52.6)	259781 (54.1)
Regular consumption of foods, *n* (%)			
Fresh fruit	45066 (22.8)	90100 (31.9)	135166 (28.2)
Fresh vegetable	194270 (98.2)	277621 (98.3)	471891 (98.3)
Fish	18389 (9.3)	23819 (8.4)	42208 (8.8)
Prior diseases, *n* (%)			
CHD	4801 (2.4)	7866 (2.8)	12667 (2.6)
Stroke or TIA	4174 (2.1)	3107 (1.1)	7281 (1.5)
Cancer	859 (0.43)	1412 (0.50)	2271 (0.47)
Family history of diabetes, *n* (%)	8600 (4.3)	13355 (4.7)	21955 (4.6)
BMI (kg/m^2)			
Mean	23.4 (3.2)	23.7 (3.4)	23.6 (3.3)
<24 (normal), *n* (%)	117507 (59.4)	157613 (55.8)	275120 (57.3)
24-28 (overweight), *n* (%)	63597 (32.2)	93414 (33.1)	157011 (32.7)
≥28 (obese), *n* (%)	16632 (8.4)	31390 (11.1)	48022 (10.0)
Physical activity, Met-hr/day	22.4 (15.3)	20.8 (12.8)	21.5 (13.9)
Systolic blood pressure, mm Hg	132 (19.8)	129.0 (21.6)	130.4 (20.9)
Diastolic blood pressure, mm Hg	79.1 (11.3)	76.6 (10.8)	77.6 (11.1)
Waist circumference, cm	81.7 (9.6)	78.7 (9.3)	79.9 (9.6)
Fat, %	21.9 (6.2)	31.9 (7.0)	27.8 (8.3)

Values are shown as n (%) or mean (SD).

ESM Table 6. Sex specific characteristics of CKB participants included for prospective analyses by educational level

Variable	Highest educational level attained								
	Men (n=197736)					Women (n=282417)			
	No formal school (n=17666)	Primary school (n=66325)	Middle or high school (n=98619)	College or above (n=15126)		No formal school (n=70311)	Primary school (n=88341)	Middle or high school (n=111004)	College or above (n=12761)
Age, years	60.2 (9.9)	55.7 (10.1)	48.4 (9.7)	50.2 (12.2)		56.5 (10.1)	51.4 (10.2)	46.4 (8.5)	46.1 (10.1)
Urban residence, *n* (%)	3307 (18.7)	16317 (24.6)	50568 (51.3)	13148 (86.9)		20664 (29.4)	24183 (27.4)	66679 (60.1)	11707 (91.7)
Geographic area, *n* (%)									
Eastern	9006 (51.0)	21882 (33.0)	32802 (33.3)	4515 (29.8)		35081 (49.9)	24654 (27.9)	33077 (29.8)	3067 (24.0)
Northeastern	268 (1.5)	1507 (2.3)	13040 (13.2)	5748 (38.0)		1715 (2.4)	3330 (3.8)	19723 (17.8)	6123 (48.0)
Central	1762 (10.0)	22879 (34.5)	26903 (27.3)	830 (5.5)		8639 (12.3)	31920 (36.1)	24422 (22.0)	410 (3.2)
Western	6630 (37.5)	20057 (30.2)	25874 (26.2)	4033 (26.7)		24876 (35.4)	28437 (32.2)	33782 (30.4)	3161 (24.8)
Household income (yuan/year), *n* (%)									
<10,000	8446 (47.8)	23426 (35.3)	19921 (20.2)	361 (2.4)		31267 (44.5)	30342 (34.3)	22521 (20.3)	270 (2.1)
10,000-19,999	3313 (18.8)	18890 (28.5)	31592 (32.0)	2140 (14.1)		15403 (21.9)	28534 (32.3)	37622 (33.9)	1598 (12.5)
20,000-34,999	3378 (19.1)	14010 (21.1)	27739 (28.1)	4864 (32.2)		13427 (19.1)	19097 (21.6)	31528 (28.4)	4315 (33.8)
≥35,000	2529 (14.3)	9999 (15.1)	19367 (19.6)	7761 (51.3)		10214 (14.5)	10368 (11.7)	19333 (17.4)	6578 (51.5)
Regular alcohol consumption, *n* (%)	6201 (35.1)	27804 (41.9)	46789 (47.4)	7374 (48.8)		1992 (2.8)	3187 (3.6)	4904 (4.4)	1157 (9.1)
Regular smoking, *n* (%)									
Active	11472 (64.9)	43398 (65.4)	61001 (61.9)	6146 (40.6)		2162 (3.1)	2641 (3.0)	1619 (1.5)	87 (0.7)
Passive	8282 (46.9)	37379 (56.4)	58214 (59.0)	7256 (48.0)		32710 (46.5)	50093 (56.7)	60427 (54.4)	5420 (42.5)
Regular consumption of foods, *n* (%)									
Fresh fruit	2106 (11.9)	9090 (13.7)	25870 (26.2)	8000 (52.9)		10781 (15.3)	18273 (20.7)	50924 (45.9)	10122 (79.3)
Fresh vegetable	16854 (95.4)	65226 (98.3)	97176 (98.5)	15014 (99.3)		67709 (96.3)	87140 (98.6)	110081 (99.2)	12691 (99.5)

Fish	849 (4.8)	4129 (6.2)	10961 (11.1)	2450 (16.2)		4367 (6.2)	5648 (6.4)	12080 (10.9)	1724 (13.5)
Prior diseases, *n* (%)									
CHD	331 (1.9)	1570 (2.4)	2140 (2.2)	760 (5.0)		1624 (2.3)	2527 (2.9)	3113 (2.8)	602 (4.7)
Stroke or TIA	421 (2.4)	1466 (2.2)	1883 (1.9)	404 (2.7)		839 (1.2)	1042 (1.2)	1076 (1.0)	150 (1.2)
Cancer	95 (0.54)	348 (0.52)	339 (0.34)	77 (0.51)		310 (0.44)	461 (0.52)	537 (0.48)	104 (0.82)
Family history of diabetes, *n* (%)	219 (1.2)	1325 (2.0)	5759 (5.8)	1297 (8.6)		1087 (1.5)	2244 (2.5)	8292 (7.5)	1732 (13.6)
BMI (kg/m^2)									
Mean	22.2 (3.0)	22.8 (3.1)	23.7 (3.2)	24.6 (3.1)		23.6 (3.5)	23.8 (3.5)	23.8 (3.3)	23.2 (3.0)
<24 (normal), *n* (%)	13077 (74.1)	44628 (67.3)	53633 (54.4)	6169 (40.8)		39981 (56.9)	48507 (54.9)	61058 (55.0)	8067 (63.2)
24-28 (overweight), *n* (%)	3875 (21.9)	17818 (26.9)	35005 (35.5)	6899 (45.6)		22527 (32.0)	29407 (33.3)	37706 (34.0)	3774 (29.6)
≥28 (obese), *n* (%)	714 (4.0)	3879 (5.8)	9981 (10.1)	2058 (13.6)		7803 (11.1)	10427 (11.8)	12240 (11.0)	920 (7.2)
Physical activity, Met-hr/day	24.0 (16.3)	22.5 (16.0)	23.0 (15.2)	15.9 (9.2)		22.4 (13.8)	20.6 (13.0)	20.3 (12.4)	18.1 (8.0)
Systolic blood pressure, mm Hg	138.2 (22.4)	134.3 (20.7)	130.5 (18.6)	129.1 (18.0)		137.0 (23.2)	131.2 (21.4)	123.6 (18.8)	116.9 (17.5)
Diastolic blood pressure, mm Hg	79.8 (11.5)	78.8 (11.3)	79.1 (11.3)	78.9 (11.0)		78.4 (11.1)	77.1 (10.8)	75.5 (10.5)	73.0 (10.1)
Waist circumference, cm	77.8 (9.1)	79.7 (9.3)	83.1 (9.5)	86.3 (8.8)		79.1 (10.0)	79.3 (9.6)	78.2 (8.8)	76.3 (8.2)
Fat, %	19.3 (6.0)	20.6 (6.0)	22.8 (6.1)	24.1 (5.7)		31.5 (7.4)	32.2 (7.2)	32.1 (6.7)	30.8 (6.1)

Values are shown as n (%) or mean (SD).

ESM Table 7. Sex specific characteristics of CKB participants included for prospective analyses by household income

Variable	Household income (yuan/year)								
	Men (n=197736)					Women (n=282417)			
	<10000 (n=52154)	10000-19999 (n=55935)	20000-34999 (n=49991)	≥35000 (n=39656)		<10000 (n=84400)	10000-19999 (n=83157)	20000-34999 (n=68367)	≥35000 (n=46493)
Age, years	54.2 (11.4)	51.9 (10.8)	51.2 (10.4)	50.6 (10.4)		51.7 (11.3)	50.2 (10.2)	49.7 (9.7)	49.8 (9.7)
Urban residence, *n* (%)	8923 (17.1)	22751 (40.7)	27555 (55.1)	24111 (60.8)		18926 (22.4)	36969 (44.5)	38580 (56.4)	28758 (61.9)
Geographic area, *n* (%)									
Eastern	6423 (12.3)	13157 (23.5)	24386 (48.8)	24239 (61.1)		11733 (13.9)	20663 (24.8)	34176 (50.0)	29307 (63.0)
Northeastern	1873 (3.6)	6720 (12.0)	6850 (13.7)	5120 (12.9)		4544 (5.4)	10453 (12.6)	9410 (13.8)	6484 (13.9)
Central	15523 (29.8)	20154 (36.0)	11361 (22.7)	5336 (13.5)		18813 (22.3)	26845 (32.3)	14243 (20.8)	5490 (11.8)
Western	28335 (54.3)	15904 (28.4)	7394 (14.8)	4961 (12.5)		49310 (58.4)	25196 (30.3)	10538 (15.4)	5212 (11.2)
Highest educational level, *n* (%)									
No formal school	8446 (16.2)	3313 (5.9)	3378 (6.8)	2529 (6.4)		31267 (37.0)	15403 (18.5)	13427 (19.6)	10214 (22.0)
Primary school	23426 (44.9)	18890 (33.8)	14010 (28.0)	9999 (25.2)		30342 (36.0)	28534 (34.3)	19097 (27.9)	10368 (22.3)
Middle or high school	19921 (38.2)	31592 (56.5)	27739 (55.5)	19367 (48.8)		22521 (26.7)	37622 (45.2)	31528 (46.1)	19333 (41.6)
College or above	361 (0.7)	2140 (3.8)	4864 (9.7)	7761 (19.6)		270 (0.32)	1598 (1.9)	4315 (6.3)	6578 (14.1)
Regular alcohol consumption, *n* (%)	19070 (36.6)	25162 (45.0)	24111 (48.2)	19825 (50.0)		3296 (3.9)	3082 (3.7)	2707 (4.0)	2155 (4.6)
Regular smoking, *n* (%)									
Active	33914 (65.0)	34038 (60.9)	30230 (60.5)	23835 (60.1)		3277 (3.9)	1895 (2.3)	859 (1.3)	478 (1.0)
Passive	26502 (50.8)	31716 (56.7)	28995 (58.0)	23918 (60.3)		45154 (53.5)	45212 (54.4)	35147 (51.4)	23137 (49.8)
Regular consumption of foods, *n* (%)									
Fresh fruit	5923 (11.4)	11628 (20.8)	14054 (28.1)	13461 (33.9)		13986 (16.6)	26136 (31.4)	27521 (40.3)	22457 (48.3)
Fresh vegetable	50033 (95.9)	55289 (98.8)	49584 (99.2)	39364 (99.3)		81002 (96.0)	82435 (99.1)	67924 (99.4)	46260 (99.5)
Fish	1799 (3.4)	4185 (7.5)	5824 (11.7)	6581 (16.6)		3114 (3.7)	6117 (7.4)	7193 (10.5)	7395 (15.9)

Prior diseases, *n* (%)									
CHD	986 (1.9)	1513 (2.7)	1366 (2.7)	936 (2.4)		1970 (2.3)	2571 (3.1)	2019 (3.0)	1306 (2.8)
Stroke or TIA	1263 (2.4)	1347 (2.4)	978 (2.0)	586 (1.5)		1009 (1.2)	1020 (1.2)	656 (1.0)	422 (0.9)
Cancer	273 (0.52)	237 (0.42)	189 (0.38)	160 (0.40)		400 (0.47)	441 (0.53)	358 (0.52)	213 (0.46)
Family history of diabetes, *n* (%)	1283 (2.5)	2373 (4.2)	2671 (5.3)	2273 (5.7)		2243 (2.7)	3967 (4.8)	4052 (5.9)	3093 (6.7)
BMI (kg/m^2)									
Mean	22.5 (3.0)	23.4 (3.2)	23.7 (3.2)	24.1 (3.2)		23.5 (3.5)	23.9 (3.5)	23.7 (3.3)	23.7 (3.2)
<24 (normal), *n* (%)	37138 (71.2)	33462 (59.8)	27379 (54.8)	19528 (49.2)		48891 (57.9)	44597 (53.6)	38234 (55.9)	25891 (55.7)
24-28 (overweight), *n* (%)	12450 (23.9)	17679 (31.6)	17797 (35.6)	15671 (39.5)		26395 (31.3)	28263 (34.0)	22914 (33.5)	15842 (34.1)
≥28 (obese), *n* (%)	2566 (4.9)	4794 (8.6)	4815 (9.6)	4457 (11.2)		9114 (10.8)	10297 (12.4)	7219 (10.6)	4760 (10.2)
Physical activity, Met-hr/day	22.0 (16.2)	21.6 (15.7)	23.4 (14.8)	22.8 (14.1)		20.7 (12.4)	19.3 (12.3)	21.9 (13.5)	21.9 (13.1)
Systolic blood pressure, mm Hg	133.3 (21.1)	131.9 (19.7)	132.2 (19.3)	131.9 (18.8)		131.2 (22.6)	128.5 (21.4)	128.1 (20.9)	127.3 (20.6)
Diastolic blood pressure, mm Hg	78.4 (11.4)	78.7 (11.4)	79.4 (11.2)	80.0 (11.1)		76.8 (11.1)	76.5 (10.9)	76.6 (10.7)	76.5 (10.5)
Waist circumference, cm	79.2 (9.0)	81.8 (9.7)	82.6 (9.7)	83.8 (9.6)		78.6 (9.6)	79.1 (9.5)	78.4 (9.1)	78.5 (8.8)
Fat, %	20.1 (6.0)	21.8 (6.1)	22.5 (6.1)	23.4 (6.1)		31.5 (7.3)	32.3 (7.1)	31.9 (6.8)	32.0 (6.7)

Values are shown as n (%) or mean (SD).

ESM Table 8. Adjusted HRs and 95% CIs for incident diabetes associated with educational level in men and women

Educational level	Model 1		Model 2		Model 3	
	HR (95% CI)	p	HR (95% CI)	p	HR (95% CI)	p
Men						
No formal school	1		1		1	
Primary school	1.01 (0.90, 1.13)	0.84	0.97 (0.87, 1.08)	0.56	0.94 (0.84, 1.05)	0.26
Middle and high school	1.08 (0.96, 1.23)	0.21	1.00 (0.88, 1.13)	0.95	0.94 (0.83, 1.07)	0.36
College or above	1.27 (1.07, 1.51)	0.0076	1.15 (0.97, 1.37)	0.12	1.11 (0.93, 1.32)	0.24
p for departure for a linear trend[a]	0.26		0.13		0.035	
Women						
No formal school	1		1		1	
Primary school	0.95 (0.89, 1.02)	0.18	0.94 (0.87, 1.00)	0.066	0.94 (0.87, 1.01)	0.075
Middle and high school	0.79 (0.72, 0.87)	<0.0001	0.82 (0.75, 0.90)	<0.0001	0.85 (0.78, 0.94)	0.00062
College or above	0.80 (0.67, 0.95)	0.010	0.91 (0.76, 1.09)	0.30	0.99 (0.83, 1.19)	0.92
p for departure for a linear trend[a]	0.047		0.072		0.042	

Model 1: stratified by age at baseline (5-year age group) and study regions, and adjusted for age at baseline (continuous), family history of diabetes and household income.

Model 2: model 1 plus BMI (continuous).

Model 3: model 2 plus waist circumference, fat percentage, physical activity, regular alcohol consumption, active smoking, passive smoking, regular consumption of fish, regular consumption of fresh vegetable, regular consumption of fresh fruit, CHD, stroke or TIA, cancer, systolic blood pressure and diastolic blood pressure.

[a]Values≥0.05 suggests that the trend is linear.

ESM Table 9. Adjusted HRs and 95% CIs for incident diabetes associated with household income in men and women

Household income (yuan/year)	Model 1		Model 2		Model 3	
	HR (95% CI)	p	HR (95% CI)	p	HR (95% CI)	p
Men						
<10,000	1		1		1	
10,000-19,999	1.03 (0.92, 1.15)	0.64	0.93 (0.83, 1.04)	0.18	0.92 (0.82, 1.03)	0.14
20,000-34,999	1.13 (1.00, 1.28)	0.05	0.98 (0.87, 1.11)	0.78	0.96 (0.85, 1.08)	0.48
≥35,000	1.36 (1.19, 1.55)	<0.0001	1.07 (0.94, 1.22)	0.30	1.01 (0.88, 1.16)	0.87
p for departure for a linear trend[a]	0.092		0.066		0.14	
Women						
<10,000	1		1		1	
10,000-19,999	1.03 (0.95, 1.12)	0.48	0.99 (0.91, 1.07)	0.75	1.00 (0.92, 1.08)	0.92
20,000-34,999	1.06 (0.96, 1.16)	0.24	1.00 (0.92, 1.10)	0.92	1.01 (0.92, 1.11)	0.88
≥35,000	1.06 (0.95, 1.17)	0.28	0.97 (0.87, 1.07)	0.55	0.96 (0.87, 1.07)	0.50
p for departure for a linear trend[a]	0.87		0.69		0.64	

Model 1: stratified by age at baseline (5-year age group) and study regions, and adjusted for age at baseline (continuous), family history of diabetes, educational level and household size.

Model 2: model 1 plus BMI (continuous).

Model 3: model 2 plus waist circumference, fat percentage, physical activity, regular alcohol consumption, active smoking, passive smoking, regular consumption of fish, regular consumption of fresh vegetable, regular consumption of fresh fruit, CHD, stroke or TIA, cancer, systolic blood pressure and diastolic blood pressure.

[a]Values≥0.05 suggests that the trend is linear.

ESM Table 10. Sex specific incidence and HRs (95% CIs) for incident diabetes associated with composite measure of educational level and household income

Composite measure of SES	No. of events	Crude incidence rate (no./1000 person-years)	Standardised[a] incidence rate (no./1000 person-years)	HR (95% CI)[b]	p
Men					
Low education and low income	925	2.44 (2.29, 2.61)	2.06 (1.91, 2.22)	1	
Low education and high income	897	4.35 (4.07, 4.64)	4.01 (3.72, 4.37)	1.15 (1.02, 1.29)	0.020
High education and low income	657	1.67 (1.55, 1.80)	1.95 (1.79, 2.11)	1.04 (0.93, 1.17)	0.47
High education and high income	1128	2.72 (2.56, 2.88)	3.04 (2.86, 3.24)	1.33 (1.20, 1.48)	<0.0001
Women					
Low education and low income	2326	3.04 (2.92, 3.17)	2.58 (2.47, 2.70)	1	
Low education and high income	1949	5.19 (4.97, 5.43)	7.10 (6.58, 7.67)	1.03 (0.95, 1.11)	0.48
High education and low income	732	1.60 (1.49, 1.72)	2.43 (2.21, 2.68)	0.82 (0.75, 0.91)	0.0001
High education and high income	930	2.16 (2.02, 2.30)	2.52 (2.35, 2.70)	0.86 (0.78, 0.94)	0.0013

[a]5-year age group standardised to the whole CKB population.

[b]Stratified by age at baseline (5-year age group) and study regions, and adjusted for age at baseline (continuous), family history of diabetes and household size.

ESM Table 11. Adjusted ORs (95% CIs) for baseline prevalent diabetes and HRs (95% CIs) for incident diabetes associated with educational level in men and women without any CHD, stroke or TIA, or cancer at baseline

Educational level	OR (95% CI)	p	HR (95% CI)	p
Men				
No formal school	1		1	
Primary school	1.24 (1.14, 1.35)	<0.0001	1.01 (0.90, 1.14)	0.83
Middle and high school	1.36 (1.25, 1.49)	<0.0001	1.09 (0.96, 1.24)	0.21
College or above	1.20 (1.07, 1.34)	0.0017	1.30 (1.08, 1.56)	0.0062
p for departure for a linear trend[a]	<0.0001		0.22	
Women				
No formal school	1		1	
Primary school	1.17 (1.12, 1.22)	<0.0001	0.94 (0.87, 1.01)	0.082
Middle and high school	0.94 (0.89, 1.00)	0.035	0.78 (0.71, 0.86)	<0.0001
College or above	0.65 (0.58, 0.72)	<0.0001	0.77 (0.63, 0.93)	0.0083
p for departure for a linear trend[a]	<0.0001		0.12	

Logistic regression models were adjusted for age at baseline (continuous), study regions, family history of diabetes and household income.

Cox regression models were stratified by age at baseline (5-year age group) and study regions, and adjusted for age at baseline (continuous), family history of diabetes and household income.

[a]Values≥0.05 suggests that the trend is linear.

ESM Table 12. Adjusted ORs (95% CIs) for baseline prevalent diabetes and HRs (95% CIs) for incident diabetes associated with household income in men and women without any CHD, stroke or TIA, or cancer at baseline

Household income (yuan/year)	OR (95% CI)	p	HR (95% CI)	p
Men				
<10,000	1		1	
10,000-19,999	1.20 (1.12, 1.28)	<0.0001	1.01 (0.90, 1.14)	0.85
20,000-34,999	1.27 (1.18, 1.37)	<0.0001	1.11 (0.97, 1.26)	0.12
≥35,000	1.44 (1.33, 1.56)	<0.0001	1.33 (1.16, 1.53)	<0.0001
p for departure for a linear trend[a]	0.068		0.064	
Women				
<10,000	1		1	
10,000-19,999	1.16 (1.10, 1.21)	<0.0001	1.02 (0.93, 1.11)	0.73
20,000-34,999	1.20 (1.13, 1.27)	<0.0001	1.05 (0.95, 1.15)	0.36
≥35,000	1.28 (1.20, 1.36)	<0.0001	1.07 (0.96, 1.19)	0.22
p for departure for a linear trend[a]	0.0089		0.98	

Logistic regression models were adjusted for age at baseline (continuous), study regions, family history of diabetes, educational level and household size.

Cox regression models were stratified by age at baseline (5-year age group) and study regions, and adjusted for age at baseline (continuous), family history of diabetes, educational level and household size.

[a]Values≥0.05 suggests that the trend is linear.

ESM Fig. 1. Flowchart for selection of participants included in the analyses

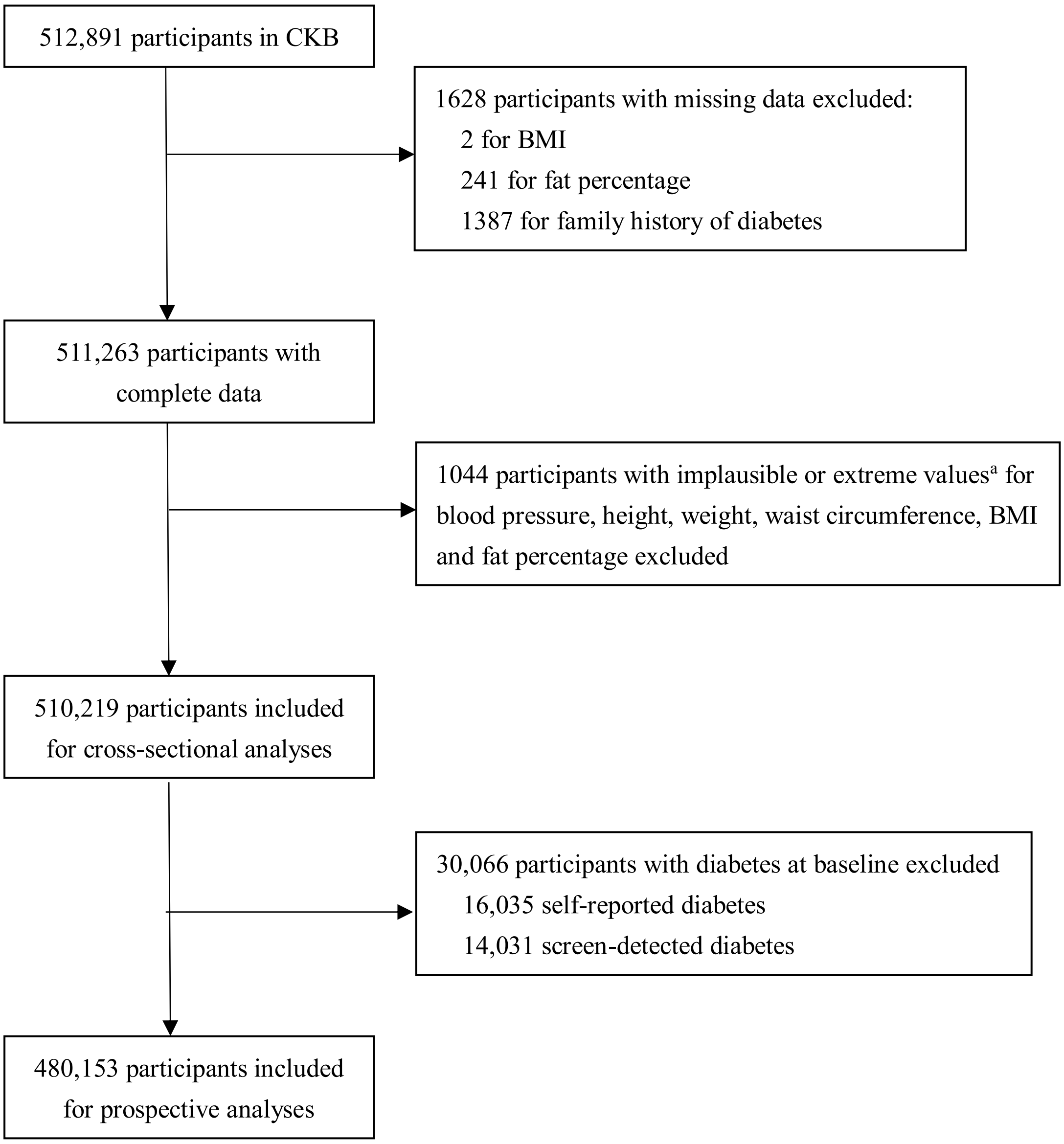

[a]Values out of 0.01 and 99.99 percentiles of the distribution.

ESM Fig. 2: Adjusted HRs and 95% CIs for incident diabetes comparing those with the highest versus lowest educational level in men and women according to baseline characteristics

Subgroup	Men	No. of event	HR (95% CI)	p for interaction	Women	No. of event	HR (95% CI)	p for interaction
Age (yr)				0·90				0·89
30-49		1057	1·02 (0·69, 1·50)			1668	0·76 (0·56, 1·03)	
50-59		1253	1·34 (0·97, 1·84)			2301	0·78 (0·57, 1·08)	
60-79		1297	1·37 (1·06, 1·77)			1968	0·87 (0·63, 1·21)	
Residence				0·21				0·83
Urban		1512	1·17 (0·88, 1·56)			2414	0·84 (0·68, 1·04)	
Rural		2095	0·95 (0·61, 1·46)			3523	0·77 (0·42, 1·41)	
Geographic areas				0·063				0·18
Eastern		1706	1·07 (0·79, 1·44)			2994	0·79 (0·54, 1·16)	
Northeastern		423	1·53 (0·55, 4·22)			612	0·94 (0·63, 1·41)	
Central		563	0·90 (0·38, 2·14)			875	0·86 (0·35, 2·15)	
Western		915	1·07 (0·75, 1·52)			1456	0·62 (0·41, 0·94)	
Household income (yuan/yr)				0·68				0·65
<10000		747	1·43 (0·61, 3·34)			1522	0·69 (0·22, 2·21)	
10000-19999		835	1·10 (0·71, 1·70)			1536	0·80 (0·50, 1·27)	
20000-34999		1003	1·31 (0·94, 1·82)			1650	0·79 (0·58, 1·08)	
≥35000		1022	1·00 (0·73, 1·36)			1229	0·70 (0·51, 0·95)	
**Overall**		**3607**	**1·27 (1·07, 1·51)**			**5937**	**0·80 (0·67, 0·95)**	
	0·20 0·50 1·0 2·0 5·0				0·20 0·50 1·0 2·0 5·0			

Cox models were stratified by age at baseline (5-year age group) and study regions, and adjusted for age at baseline (continuous), family history of diabetes and household income as appropriate.

ESM Fig. 3: Adjusted HRs and 95% CIs for incident diabetes comparing those with the highest versus lowest household income in men and women according to baseline characteristics

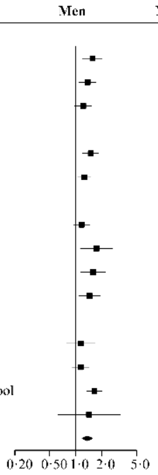

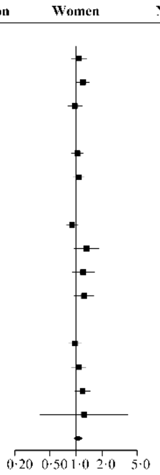

Subgroup	Men: No. of event	Men: HR (95% CI)	Men: p for interaction	Women: No. of event	Women: HR (95% CI)	Women: p for interaction
Age (yr)			0·18			0·11
30-49	1057	1·56 (1·20, 2·02)		1668	1·08 (0·88, 1·33)	
50-59	1253	1·37 (1·09, 1·72)		2301	1·20 (1·01, 1·42)	
60-79	1297	1·22 (0·98, 1·52)		1968	0·97 (0·81, 1·17)	
Residence			0·035			0·99
Urban	1512	1·49 (1·20, 1·83)		2414	1·04 (0·89, 1·21)	
Rural	2095	1·26 (1·05, 1·50)		3523	1·07 (0·93, 1·23)	
Geographic areas			0·017			0·010
Eastern	1706	1·17 (0·95, 1·45)		2994	0·90 (0·78, 1·05)	
Northeastern	423	1·73 (1·13, 2·67)		612	1·32 (0·96, 1·82)	
Central	563	1·58 (1·14, 2·18)		875	1·21 (0·90, 1·63)	
Western	915	1·44 (1·08, 1·92)		1456	1·24 (0·95, 1·61)	
Educational level			0·68			0·65
No formal school	480	1·15 (0·79, 1·67)		2330	0·97 (0·82, 1·15)	
Primary school	1342	1·14 (0·91, 1·42)		1945	1·08 (0·89, 1·31)	
Middle or high school	1469	1·64 (1·33, 2·02)		1485	1·19 (0·97, 1·46)	
College or above	316	1·42 (0·62, 3·23)		177	1·24 (0·39, 3·96)	
**Overall**	**3607**	**1·36 (1·19, 1·55)**		**5937**	**1·06 (0·95, 1·17)**	

Cox models were stratified by age at baseline (5-year age group) and study regions, and adjusted for age at baseline (continuous), family history of diabetes, household size and educational level as appropriate.
